# Death by Chloroform

**Published:** 1852-09

**Authors:** 


*Death by Chloroform.*—I have this moment received a note from you requesting an account of the unfortunate case of etherization that occurred in my practice on Friday last. It was my intention to have drawn up a statement of the case for your Journal, and so will at once comply with your request.

Henry Keyser, a German by birth, aged 17, was brought to my office yesterday about noon for surgical aid. The middle finger of his left hand had been caught in the gearing of the machine at which he was at work; the last phalanx was carried away and the soft parts badly lacerated and torn. It was thought best to remove a portion of the next phalanx, so that when the mangled muscles, ligaments, &c., should be removed, the extremity of the bone might still be covered. The young man looked pale, and appeared to suffer great pain; but beyond this I observed nothing unusual in his appearance—and saw no reason why ether might not safely be administered, and the further infliction of pain obviated. I made the proposition, and he at once began to inhale a mixture of chloric ether and chloroform. There was about ten per cent. of chloroform contained in the mixture. The inhalation was continued for about four or five minutes, with now and then an interruption by his pushing the sponge away with his hand. Appearing insensible, the operation was commenced, but the first stroke of the knife made him start with a loud expression of pain. He began to vomit, and now sensibility was in a great measure restored. The sponge, replenished, was again applied, and he complained of its making his lips smart. I at once removed it and applied ol. oliv. The sponge was again applied over the nose and mouth, and inhalation carried on as before, and for about the same length of time as at the first attempt, or it may have been a minute or two longer. He again appeared unconscious; and to prevent his waking up as before, I gave the sponge—now containing very little ether—into the hands of Mr. Merrill, directing him to hold it still over the face; but there was again retching and an attempt to vomit, from which cause he but illy succeeded in retaining the sponge as directed. I again commenced the operation, Mr. Venner, who came with the young man, holding the hand.

Up to this time, there was observed nothing remarkable either in his general appearance, the pulse, or the respiration. I had made only two incisions, and was attempting to tie the outside digital artery, which was bleeding, per saltem, showing that up to this moment the circulation was good, when my attention was directed by Mr. Merrill to the appearance of the patient.

I saw at once that he was either dead or dying, and directed my assistants to help me lay him at once on his back. I sent one of them for medical counsel, whilst the other assisted me in applying restoratives. I found the pulse at the wrist gone, the action of the heart very feeble indeed, and respiration in a moment ceased. But by the application of strong ammonia to the nose, dashing ice-water over the head, &c., he again began to gasp, and was breathing convulsively when Dr. Parcher arrived. But a few heaving inspirations, at long intervals—the action of the heart meanwhile growing more and more feeble—and all was quiet. My patient was dead. Dr. Parcher assisted me in the diligent application of the usual means for resuscitation in cases of suspended animation—but all to no purpose, the vital spark had fled.

I regret that I am unable to give the post-mortem appearances of the internal vital organs, the friends objecting to an autopsy being made. And yet I very much doubt whether the knife would have revealed anything new. Such, then, are the facts in this unfortunate case. I had read of deaths from chloroform, but had hoped never to have seen one. A coroner's inquest was held at my request over the body of the deceased, a few hours after death, and the jury returned a verdict in accordance with the facts above stated.

But the question will arise in the minds of all who may read this article, how was death caused in this particular case? Was it owing to an impure article used? Was it unskillfully administered? Or was there something peculiar in the organization of the patient, or the state of the nervous system at the time, rendering this agent toxical with whatever precaution used? That the last position is the true one, is my honest conviction.

On the first question, I have only to remark, that both the chloric ether and chloroform were obtained at W. B. Little's apothecary store, on Hanover street, where I have usually supplied myself for several years past. And in addition, I can state this important fact, that from the same bottle I had administered the ether in a number of cases previously, with the usual effect. And furthermore, the mixture in the phial, containing the small per cent. of chloroform mentioned, had been tested only a few days before, in a case where a similar operation on a finger was required. This patient inhaled from two to three times the quantity that Keyser did, with the happiest effect, and walked home after the operation.

Secondly, I would remark that I have habitually used anæsthetic agents in my practice, since their first introduction in this city, and never before witnessed any alarming or injurious effect from their use. At first I employed sulphuric ether; then, for nearly two years, chloroform exclusively. But finding that, according to the experience of others, ether seemed more safe, I have for almost two years relied upon this agent in obstetric and surgical practice, using chloroform only in cases where the ether appeared not to induce anæsthesia very readily. My plan of administering these agents has been uniform. I have never used an inhaler of any kind whatever. The sponge is the only article I have ever used, unless it was the handkerchief or napkin in a few instances when no sponge was at hand. I have always made it a point to admit an abundant supply of atmospheric air into the lungs, and when the patient complained of suffocation permitted him to push the sponge away for a few moments, and then go on with the inhalation. The sponge I used the other day is of small size, not holding above two fluid ounces, and was seldom filled beyond half its capacity. It is the same sponge that I have used for more than five years, and is so open, that of itself, it is no obstacle whatever to respiration when placed over the mouth and nose.

In regard to the quantity used in the present instance—the phial had on it an apothecary's label; and, before moistening the sponge, the fluid did not reach the lower edge of said label. This fact was remarked by Mr. Venner as well as myself, and there still remains in the phial nearly one ounce of the fluid. In the opinion of the physicians—Drs. Parcher and Thorndyke, who were on the inquest—the quantity used was about two ounces or a little over. By weight it may have been more.

Finally, I learned from the mother of the young man, that he had never been sick, but had been a child of penury and want, suffering at times for the necessaries of life; also, that he had met with an accident some years since, on account of which he lay in a fainting condition for some time. One of the men who came to my office with him, but who passed out as I came in, told me, the day after, that he "had no doubt but that Henry died from the effects of fear;" that he trembled like an aspen leaf when he was coming from the shop. We all know what a terrible influence *fear* has over the vital economy. Why, a friend of mine mentioned, in connection with this case, that he once knew an artisan, who died in this way without taking ether. His knife slipped in his hand, and he *thought* he had inflicted a serious wound on his thigh. He swooned away, and all efforts to revive him were unavailing. The surgeon found only a slight scratch of the skin—but his patient was dead.

My conclusion, then, is, that the fatal consequence attending etherization in the present instance is not owing to any inferiority in the article used, to want of care in its administration, nor to any organic disease in the patient; but that we must look for it in the naturally delicate organization of the subject, rendering him very sensitive to external impressions, in the shock that the nervous system had sustained in the injury, and last, but not least, in the influence of *fear*. Not in any one of these singly, but in the three combined.

In haste, yours most truly, DAN'L V. FOLTS.

37 Maverick Square, E. Boston, Aug. 9, 1852.

*Boston Med. and Surg. Journal.*

